# Persistent Gingival Bleeding Related to Periodontal Disease

**DOI:** 10.7759/cureus.77693

**Published:** 2025-01-20

**Authors:** Yosuke Iijima, Miki Yamada, Takumi Takahashi, Shunsuke Hino, Motohiko Sano, Hiroshi Sakagami, Norio Horie, Takahiro Kaneko

**Affiliations:** 1 Department of Oral and Maxillofacial Surgery, Saitama Medical Center, Saitama Medical University, Saitama, JPN; 2 Division of Applied Pharmaceutical Education and Research, Hoshi University, Tokyo, JPN; 3 Research Institute of Odontology, Meikai University, Saitama, JPN

**Keywords:** acute gingival bleeding, anticoagulant drugs, antiplatelet drugs, periodontal disease (pd), s: hypertension

## Abstract

Objective

More patients visit oral surgery outpatient clinics from evening to night to treat persistent gingival bleeding (PGB) related to periodontal disease (PD) (PD-PGB). Since there are few reports on PD-PGB, the present study performed a detailed characterisation of this disease.

Materials and methods

Patients who visited our oral surgery clinic between 1 January 2014 and 31 December 2022 to treat PD-PGB without trauma. Demographic data, systemic diseases and bleeding tooth characteristics were retrieved. The occurrence of systemic diseases was compared with that of the control group (patients consisting of temporomandibular joint dislocation and minor trauma).

Results

Among 295 patients with gingival bleeding (GB) unrelated to trauma, 193 patients (65.4%) showed postoperative bleeding, and 91 patients (30.1%) showed PD-PGB; 93.4% of patients over 60 years old showed PD-PGB. Patients in the PGB-RPD group showed a significantly higher percentage (57.1%) of taking anticoagulants and/or antiplatelets than the control group (24.2%) (P < 0.001) Significantly higher percentage of patients in the PGB-RPD group showed a significantly higher incidence of hypertension (68.1%) than the control group (34.1%) (P < 0.001). Bleeding is the most frequently observed in molars, with a depth of the pockets exceeding 4 mm.

Conclusion

PD-PGB accompanied by deep pockets was more common in older patients and aggravated by taking anticoagulants and/or antiplatelets and antihypertensives. PD-PGB is not rare, and periodontal maintenance, such as regular dental checks, is an important preventive measure.

## Introduction

Many patients with bleeding as the main complaint, as well as trauma patients, visit oral surgery outpatient clinics. Apart from trauma-mediated bleeding, gingival bleeding (GB) is the most common, and among GB, postoperative bleeding, including post-extraction bleeding, is the most frequent. However, a certain number of patients with GB also have spontaneous bleeding from the gingival sulcus. Clinically, if the GB is seen from a gingival sulcus without any abnormal findings such as trauma, it is quite reasonable to assume that the local cause of this GB is clearly related to periodontal disease (PD).

The hospital is a regional core hospital, including our department, and also offers a 24-hour outpatient clinic, where patients with GB associated with PD (PD-GB) also come to the hospital. PD-GB may seem commonplace and inconsequential, but many patients with persistent PD-GB (PD-PGB) are seen late evening and night (out-of-hours hours) rather than in the daytime. The PD-GB often starts suddenly and is persistent and prolonged due to mental stress. From the oral surgeon's point of view, gingival sulcus bleeding is more troublesome than post-extraction bleeding. This is because gingival sulcus bleeding is not suitable for haemostasis by sutures and often needs the filling of haemostatic agents, which may take longer to confirm haemostasis, especially in patients who intake anticoagulants and/or antiplatelets [1-3].

GB after tooth extraction and minor surgery has been relatively frequently reported, where anticoagulants and/or antiplatelet users, advanced age, poor oral hygiene, inferior nerve block and multiple tooth extractions are risk factors for GB [4-6]. However, to the best of our knowledge, PD-PGB has not been reported at all so far, despite the relatively high number of patients presenting to the hospital and the fact that it is a difficult condition to stop the bleeding.

The aim of this study is to clarify the actual status and risk factors for persistent PD-PGB, which has not been reported before.

## Materials and methods

This retrospective study was approved by the Research Ethics Committee of Saitama Medical Centre, Saitama Medical University (reference number: 2023-097). Patients with a chief complaint of GB other than trauma registered to the Department of Oral and Maxillofacial Surgery at the Saitama Medical Centre, Saitama Medical University, between 1 January 2014 and 31 December 2022, were monitored. From these patients, those presenting with PD-PGB were selected. The hospital, including the department, offers 24-hour out-of-hours care for emergencies as well as regular medical care. Exclusion criteria were GB not related to PD, such as postoperative bleeding, including post-extraction bleeding and other bleeding (e.g., bleeding from malignant tumours).

The examination included demographic data (age, gender), medical and medication history of anticoagulants and/or antiplatelets, time of hospital visit, time of onset of bleeding, information on the site of bleeding and PD of the tooth at the site of bleeding, treatment and the use of an ambulance. Medical history was limited to diabetes mellitus, which is closely associated with PD, and hypertension, which has been discussed in relation to PD [7]. Comparisons were made between the PD-PGB group and the control group (no GB) with regard to the medical and medication history of anticoagulants and/or antiplatelets. The control group consisted of patients who visited the department at the same time (between 1 January 2014 and 31 December 2022) without GB (diagnosis: temporomandibular joint dislocation or minor trauma) and whose age and gender were matched to the PD-PGB group. The detection date of the PD-PGB was based on the patient's report. Information on the tooth at the PD-PGB site included the location of the tooth and periodontal clinical markers (pocket depth and degree of tooth mobility). Not all cases could be examined for PD, as it is extremely difficult to accurately probe the pocket depth or measure the degree of mobility of bleeding teeth. So, for convenience, pocket depth was measured with a periodontal probe (#2, YDM, Tokyo, Japan) and a pocket depth of ≥4 mm at the bleeding site was considered to have a deep pocket. Tooth mobility was scored based on Miller’s mobility index: M1 - the first distinguishable sign of movement greater than normal; M2 - a movement of the tooth which allows the crown to move 1 mm from its normal position in any direction; M3 - allows the tooth to move more than 1 mm in any direction. Teeth that may be rotated or depressed in their alveoli are classified as mobility [8].

The treatments were divided into seven categories: A) haemostasis already on arrival; B) pressure haemostasis only; C) pressure haemostasis with local anaesthesia of surrounding gingiva; D) pressure haemostasis with haemostatic material insertion; E) pressure haemostasis with local anaesthesia of surrounding gingiva and haemostatic material insertion; F) pack application (with or without other treatment); G) suturing (with or without other treatment).

As a statistical analysis, comparisons of gender and age within the PD-PGB group were made using a binomial test. Comparisons of bleeding start time and time of visit within the PD-PGB group were made using multiple comparisons using Ryan's method. Comparison of systemic factors between PD-PGB and the control group were compared with the χ2test or Fisher’s exact test. All statistical tests were two-sided. A P-value < 0.05 was considered statistically significant. All statistical analyses were performed using js-STAR version 9 software (Hiroyuki Nakano, Joetsu University of Education, Joetsu, Japan) (http://www.kisnet.or.jp/nappa/software/star/index.htm).

## Results

A total of 295 patients with GB not resulting from trauma visited the department. These included 193 (65.4%) patients, of whom 167 (86.5%) experienced postoperative bleeding following tooth extraction. Among them, 26 (13.5%) patients had postoperative bleeding other than tooth extraction. Additionally, 91 (30.8%) patients had PD-PGB, and 11 (3.8%) experienced bleeding due to other causes, such as malignant tumours (Table 1).

**Table 1:** Classification of patients with gingival bleeding *Other bleedings were due to malignant tumours. PD-PGB, persistent gingival bleeding associated with periodontal disease.

Gingival bleeding	N (%)
Postoperative bleeding	193 (65.4)
Post-extraction bleeding	167 (86.5))
Other than post-extraction bleeding	26 (13.5))
PD-PGB	91 (30.8)
Others*	11 (3.8)
Total	295 (100)

PD-PGB group (91 patients) were 34 to 95 years old (mean ± SD, 74.4 ± 9.9). Forty-four (48.4%) patients were males, while 47 (51.6%) patients were females, with a male-to-female ratio of 1:1.07. Eighty-five (93.4%) patients were over 60 years old, which was statistically significant (P < 0.005), composed of 15 patients from 60 to 69 (17.6%) years old, 46 (54.1%) patients from 70 to 79 years old and 24 (28.2%) patients over 80 years (Table 2).

**Table 2:** Characteristics of patients with PD-PGB PD-PGB, persistent gingival bleeding associated with periodontal disease; SD, standard deviation

	PD-PGB group N (%)
Gender	
Male	44 (48.4)
Female	47 (51.6)
Age (year)	
<60	6 (6.6)
≥60	85 (93.4)
60-69	15 (17.6)
70-79	46 (54.1)
≥80	24 (28.2)
Mean ± SD	74.4 ± 9.9

Regarding the patient visit times, evening (18:00-23:59) was the most common with 50 (54.9%) cases, followed by night (0:00-05:59), 20 (22.0%) cases, morning (6:00-11:59), 12 (13.2%) cases, and then afternoon (12:00-17:59), 9 (9.0%) cases. Regarding the bleeding start time, afternoon was the most common (31 (34.1%) cases), followed by evening (30 (33.0%) cases), morning (16 (17.6%) cases) and night (14 (15.4%) cases). There was no significant difference in bleeding start time, but there was significantly more evening at the time of the visit (P < 0.001) (Table 3).

**Table 3:** Time of visit and bleeding start time of PD-PGB group PD-PGB, persistent gingival bleeding associated with periodontal disease *There was no significant difference between bleeding start time; **evening was significantly more common than the other items (P < 0.001); **P-value < 0.05.

	Bleeding start time*	Time of visit**
	N (%)	
Morning (6:00-11:59)	16 (17.6)	12 (13.2)
Afternoon (12:00-17:59)	31 (34.1)	9 (9.0)
Evening (18:00-23:59)	30 (33.0)	50 (54.9)
Night (0:00-05:59)	14 (15.4)	20 (22.0)

Significantly higher rate of use of anticoagulants and/or antiplatelets administered to 52 (57.1%) patients of the PD-PGB group, as compared with 22 (24.2%) patients of the control group (P < 0.001). The incidence of diabetes mellitus in the PD-PGB group was 17 (18.7%) patients, with no significant difference from the control group (eight (8.8%) patients) (P = 0.083). The incidence of hypertension in the PD-PGB group (62 (68.1%) patients) was significantly higher than that of the control group, 31 (34.1%) patients (P < 0.001) (Table 4).

**Table 4:** Comparison of systemic factors between PD-PGB group and control group PD-PGB, persistent gingival bleeding associated with periodontal disease *P-value < 0.05.

	PD-PGB group	Control group	P-value
	N (%)		
Anticoagulant and/or antiplatelet	52(57.1)	22 (24.2)	<0.001*
Diabetes mellitus	17 (18.7)	8 (8.8)	0.083
Hypertension	62 (68.1)	31 (34.1)	0.001*

**Figure 1:**
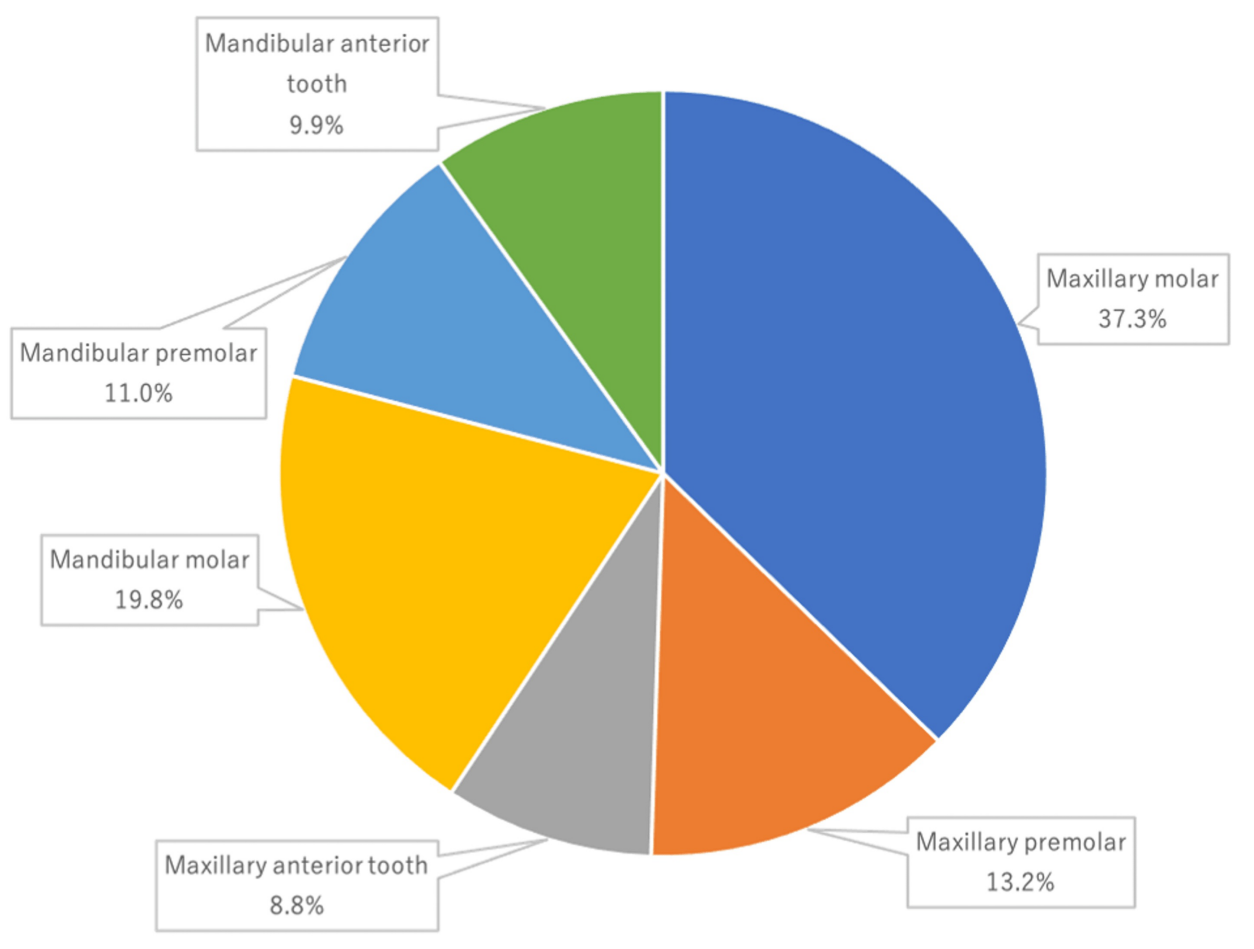
Bleeding site teeth

The main location site of the PD-PGB site was maxillary molars (34 (37.3%) cases), followed by mandibular molars (18 (19.8%) cases), maxillary premolars (12 (13.2%) cases), mandibular premolars (10 (11.0%) cases), mandibular anterior teeth (nine (9.9%) cases) and maxillary anterior teeth (eight (8.8%) cases) (Figure 1). 

**Figure 2:**
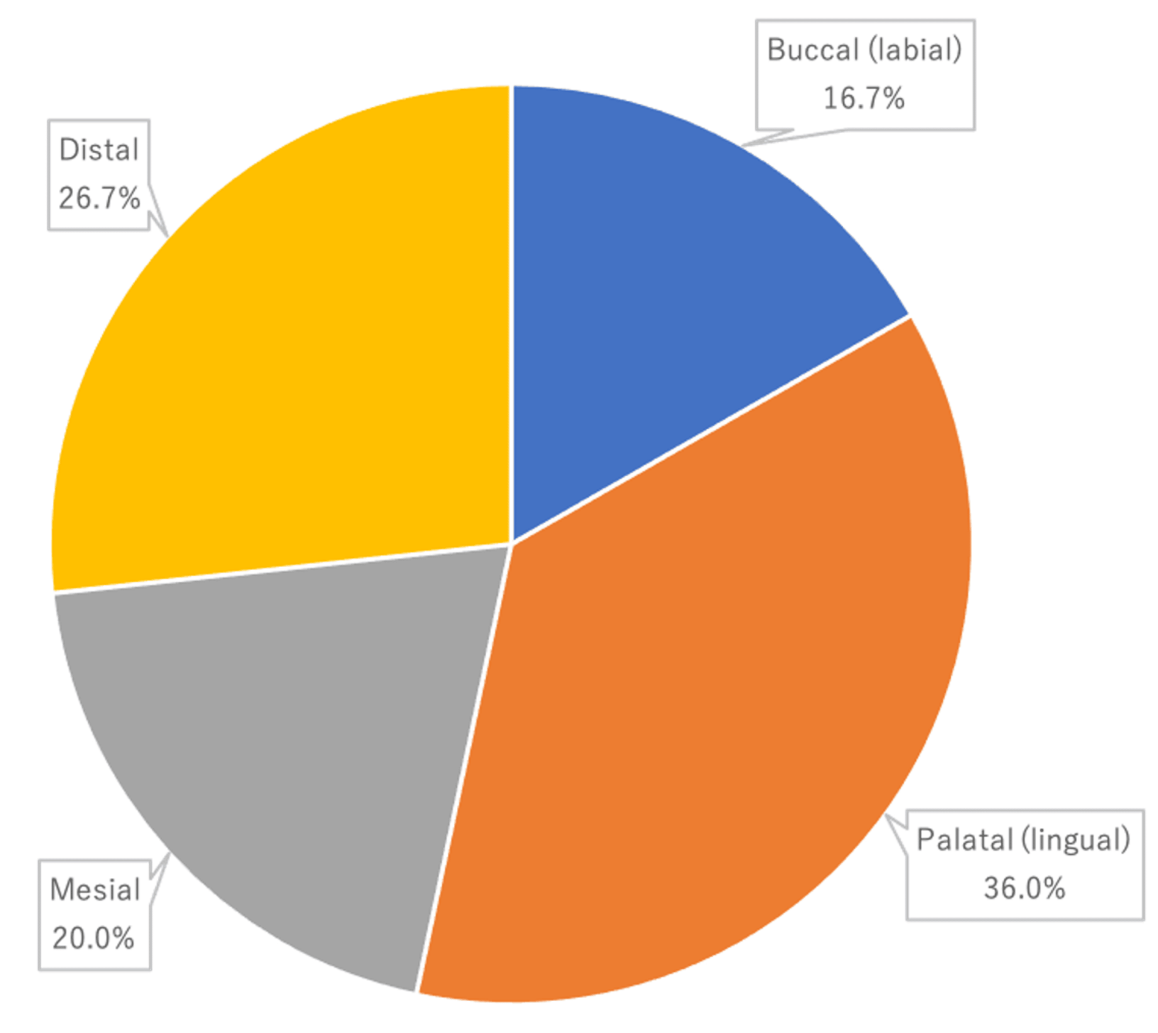
Tooth surface at the bleeding site

The most common tooth surface with detectable bleeding (30 cases) were palatal/lingual (11 (36.0%) cases), followed by distal (eight (26.7%) cases), mesial (six (20.0%)) cases and buccal/labial (five (16.7%) cases) (Figure 2).

**Figure 3:**
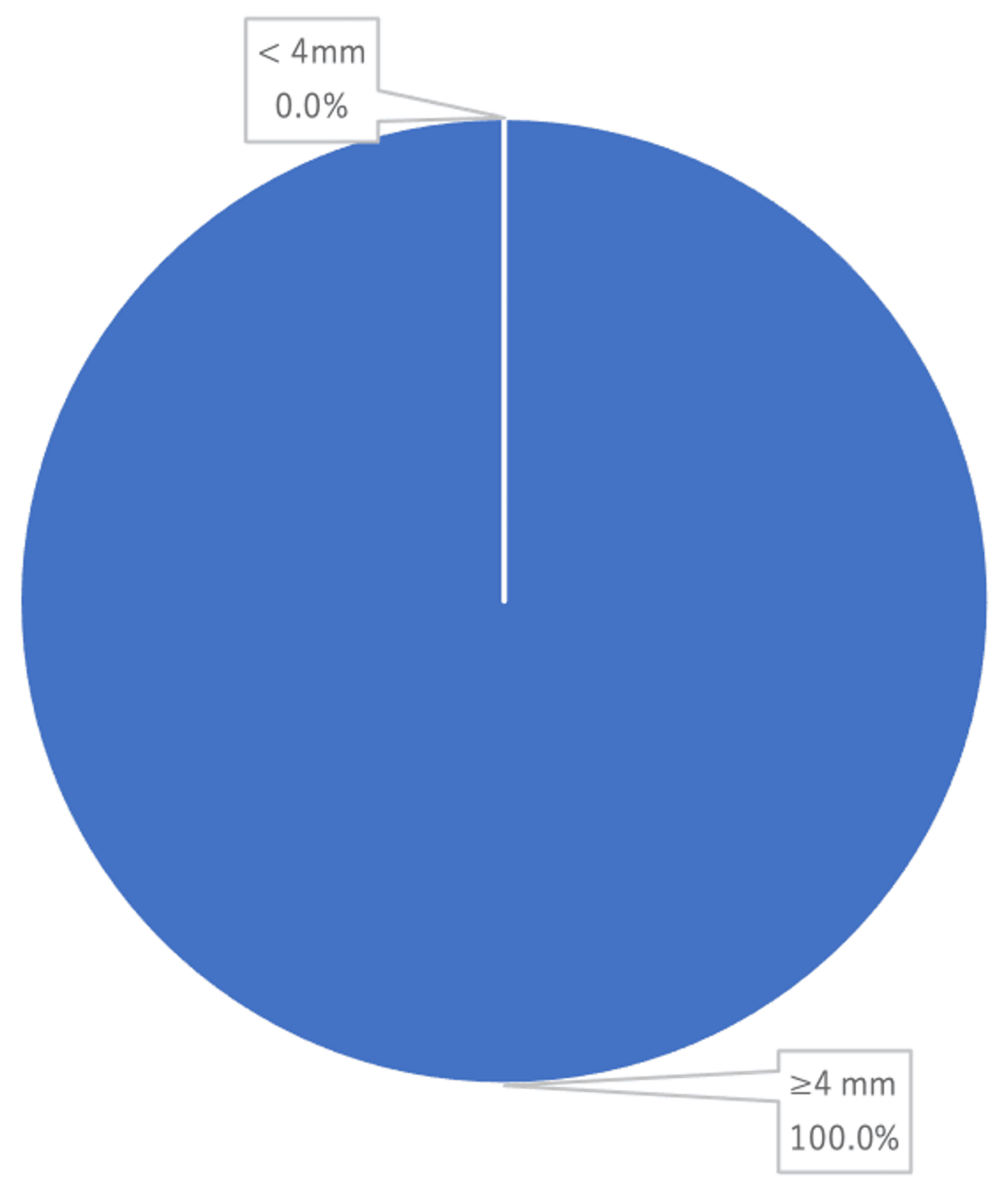
Pocket depth at the bleeding site

Pocket could be detected in 16 cases where the length of all pockets exceeded 4 mm (Figure 3).

**Figure 4:**
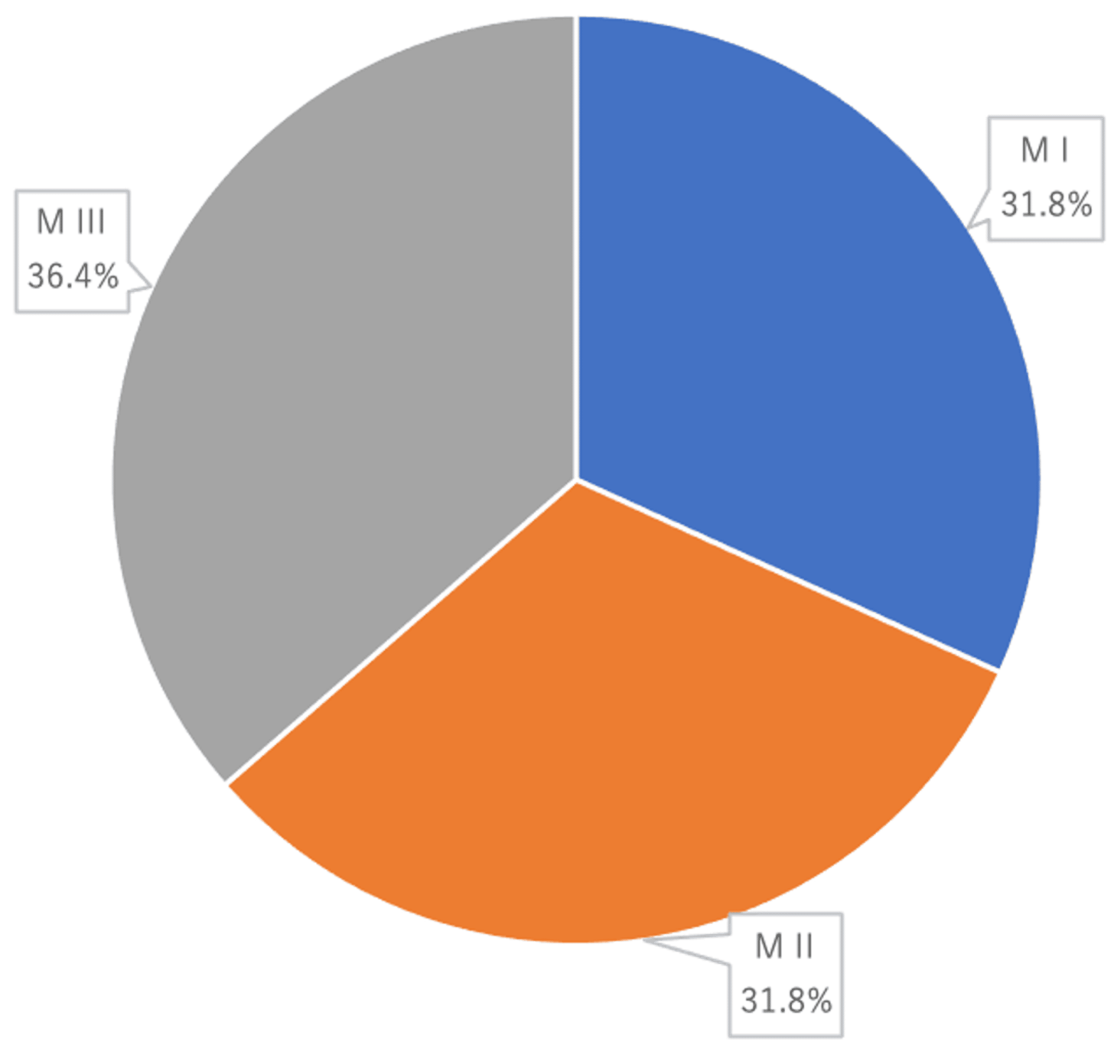
Degree of tooth mobility at the site of the bleeding

Tooth mobility could be measured in 22 cases where seven (31.8%) cases were classified as degree I, seven (31.8%) cases as degree II, and eight (36.4%) cases as degree III (Figure 4).

Treatments were as follows: A) haemostasis already on arrival was the most common (26 (28.6%) cases), B) pressure haemostasis only (19 (20.8%) cases), C) pressure haemostasis with local anaesthesia (19 (20.8%) cases), D) pressure haemostasis with haemostatic material insertion (six (6.6%) cases), E) pressure haemostasis with local anaesthesia and haemostatic material insertion (six (6.6%) cases), F) pack application (with or without other treatment) (four (4.4%) cases), G) suturing (with or without other treatment) (11 (12.1%) cases). Minor bleedings (A and B) were present in (45 (49.4%) cases). There were no cases in which the pack was fitted and sutured at the same time during the procedure (Table 5).

**Table 5:** Treatment of persistent gingival bleeding related to periodontal disease

	N (%)
A) Haemostasis already on arrival (no treatment)	26 (28.6)
B) Pressure haemostasis only	19 (20.8)
C) Pressure haemostasis with local anaesthesia	19 (20.8)
D) Pressure haemostasis with haemostatic material insertion	6 (6.6)
E) Pressure haemostasis with local anaesthesia and haemostatic material insertion	6 (6.6)
F) Pack application (with or without other treatment)	4 (4.4)
G) Suturing (with or without other treatment)	11 (12.1)
Total	91 (100)

Ambulance was used in 22 (24.2%) cases (Table 6).

**Table 6:** Use of ambulances in PD-PGB PD-PGB, persistent gingival bleeding associated with periodontal disease

Use of ambulances	N (%)
Yes	22 (24.2)
No	69 (75.8)

## Discussion

The study showed that the incidence of PD-PGB was higher in older adults and in gingival sulci with pockets depth exceeding 4 mm and increased when anticoagulants and/or antiplatelets and hypertension were used. 

Eighty-five (93.4%) of PD-PGB patients were over 60 years of age with significance (P < 0.005). Fifteen (17.6%) of them were distributed to 60- to 69-year-olds, 46 (54.1%) to 70- to 79-year-olds, and 24 (28.2%) were over 80 years old. The age-related increase in PD-PGB may be due to accompaniment with PD. However, the sharp increase in PD-PGB patients over 60 years old may be more related to the increased incidence of stroke, atrial fibrillation and other conditions requiring anticoagulants and/or antiplatelets in the 60-70 age group [9-11].

The patient visit to the hospital was more frequent during after-hours care, with 50 (54.9%) cases (evening) with significance (P < 0.001) and 20 (22.0%) cases (night). However, the most frequent bleeding start time shifted to earlier times of the day, in the afternoon (31 (34.1%) cases) and in the evening (30 (33.0%) cases), rather than the night (14 (15.4%) cases). One reason for this time shift may be that patients visit their general practitioner dentist for treatment of daytime minor bleeding. Another reason is the time lag between the patient becoming aware of the bleeding and the hospital visit. The patient will probably wait a while to see whether the bleeding stops before the hospital visit. Therefore, although patients with PD-PGB may come to the hospital late in the day, it was found that the bleeding was not concentrated in the evening or at night but was, in fact, widely distributed, with many patients bleeding in the afternoon.

Among systemic background factors, the use of anticoagulants and/or antiplatelets was significantly higher in the PD-PGB 52 (57.1%) cases compared to 22 (24.2%) cases in the control group (P < 0.001). The results suggested that the use of anticoagulants and/or antiplatelets was highly implicated in PD-PGB. Antiplatelet and anticoagulant drugs act at different sites in the coagulation system. Antiplatelet agents are usually indicated for patients with atherosclerosis. Anticoagulants are used in patients with atrial fibrillation, venous thromboembolism and valvular heart disease [12]. The typical antiplatelet drugs aspirin and clopidogrel have been reported not to increase the risk of post-extraction bleeding without withdrawal [13]. Reports on tooth extraction during anticoagulant use indicate that use of anticoagulant increases the rate of post-extraction bleeding, but only slightly when appropriate haemostatic measures are taken, and that withdrawal of anticoagulants, either warfarin or direct oral anticoagulants, is not recommended [14]. In the case of PD-PGB related to PD in this study, anticoagulants and/or antiplatelets significantly increased the bleeding tendency, but the presence of PD as a local factor should not be underestimated. Anticoagulants and/or antiplatelets have been reported to produce a small number of minor PGB as adverse events [15,16]. However, with this PGB, the extent of PD at the bleeding site has not been investigated in detail. These GB may correspond to the PD-PGB investigated in this study. 

Among reports of the relationship between PD and hypertension, there have been conflicting views on whether PD is a risk factor for hypertension [7]. Apart from this debate, there have been reports to the contrary that hypertension is a risk factor for PD. Leite et al. stated that in animal studies, experimentally induced periodontitis was exacerbated in spontaneously hypertensive rats compared to normotensive rats [17]. It was further stated that PGB could be some form of subtle marker of vessel damage beyond the setting of periodontitis, as hypertension can lead to malfunction of small arterioles [7]. Hypertension is not only a major risk factor for cardiovascular diseases such as stroke and coronary artery disease but also a systemic cause of epistaxis [18,19]. In epistaxis, bleeding from the nasal mucosa, increased blood pressure and the use of anticoagulants prolong the bleeding time [18]. In this study, hypertension was significantly more common in the PD-PGB disease group in 62 (68.1%) cases compared with 31 (34.1%) cases in the control group (P < 0.001). This suggests that hypertension may damage small vessels in periodontal tissue, but the relationship between hypertension, periodontitis, and PGB requires further research. 

Diabetes is described to be one of the risk factors for periodontitis, as supported by ample evidence [20]. This means that the impact of diabetes on immune function and inflammatory pathways may also have a negative impact on periodontal health. Recent reports suggest a bidirectional relationship between glycaemic levels and periodontitis [21]. This study showed a higher incidence of diabetes in the PD-PGB group (17 (18.7%) cases) than the control group (eight (8.8%) cases), but not significantly. Diabetes may not be a risk factor in terms of PGB, but it needs further investigation.

Localisation of the bleeding site showed that the PD-PGB was limited to a single tooth in most cases, with molars being the most common (maxillary 37.3% and mandibular 19.8%), followed by premolars (maxillary 13.2% and mandibular 11.0%) and anterior teeth (maxillary 8.8% and mandibular 9.9%). Complex root morphology (compound roots) may be involved. The majority of the PD-PGB was from only one surface, with no marked differences between the palatal/lingual, buccal/labial, mesial and distal surfaces. Incidentally, PGB in leukaemia is not limited to one tooth, and gingival hypertrophy, ulceration and necrosis may also be seen [22]. 

The search for the severity of PD was not possible in all cases, as haemostatic treatment was a priority. Although the present study focused on spontaneous bleeding from the gingival sulcus, persistent bleeding during probing is a sign of a plaque-induced inflammatory response in the pocket wall and is one of the most reliable predictors of periodontal destruction [23,24]. In those that could be measured in this search, the pocket depths exceeded 4 mm, and this suggested that the bleeding site was a deep pocket where strong inflammatory reactions occur. Tooth mobility has been considered and investigated as an indirect measure of the functional marker of the periodontium as well as the presence of aggravating co-factors for PD [25]. In this study, MI, MII and MIII were within close ranges (31.8%, 31.8% and 36.4% of the teeth, respectively), suggesting that the degree of tooth mobility may not be involved in the GB.

Twenty-six (28.6%) of the patients had already haemostasis when they arrived at the hospital, 19 (20.8%) cases needed only pressure haemostasis and 46 (50.6%) cases needed more than pressure haemostasis. Whether there is a relationship between the presence of risk factors for PD-PGB and the treatment method could not be examined due to the small number of cases in this report. Further case studies are needed to analyse this.

An ambulance was used commonly in 22 (24.2%) cases. Half of these cases were either haemostatic on arrival or pressure haemostatic (data not shown), which corresponds to minor bleeding, suggesting that patients were very surprised and concerned by the PD-PGB. 

Strategies to prevent PD-PGB are regular dental visits and treatment of PD. The treatment of patients with anticoagulants and/or antiplatelets may be troublesome, but postoperative bleeding has been reported to be infrequent, even in patients undergoing invasive periodontal treatment while taking antiplatelet or anticoagulant drugs [26]. What requires special attention from dental practitioners is the treatment of PD in the deep pockets of the molars. Diseases such as cardiovascular disease, which leads to the use of oral anticoagulants and/or antiplatelets, have been described to be associated with PD. Untreated PD may exacerbate the disease [27].

There are some limitations to this study. First, in the present study, patients with PD-PGB were seen more often during the evening and night, while the bleeding in the daytime can be seen by dentists in the city. Then, the patients seen at our hospital, which is also the regional core hospital, may be limited to severe cases, including, and there may be more minor cases of PD-PGB. Future research with a larger sample size involving multiple centres and general practitioners of dentistry could be required. Second, not all cases could be examined in detail under PGB with urgency to determine the severity of PD. Thus, it was undeniable that the number of cases could be somewhat low with regard to the examination of PD. Third, in the present study, PD-PGB was significantly more common in those with hypertension. With regard to the association between hypertension and periodontitis, it has been shown that patients with moderate to severe periodontitis are more likely to develop hypertension [28]. Patients with periodontitis show higher systolic and diastolic blood pressure values compared to patients without periodontitis [29]. Although the relationship between increased blood pressure and PD-PGB is not clear, it has been reported that increased blood pressure during antithrombotic therapy is associated with an increased risk of intracranial haemorrhage [30]. Further research is needed on the effects of hypertension and high blood pressure values on periodontitis and PD-PGB.

This study examined the risk factors for PD-PGB, the most common form of GB in out-of-hours visits other than postoperative bleeding. This information will be of great help to dental practitioners in their daily practice as knowledge for the reduction of PD-PGB that plagues their patients.

## Conclusions

In conclusion, persistent GB associated with PD was not a rare condition and was predominantly seen in older adults over 60 years old. Locally, the bleeding was from deep pockets on one surface of one tooth and was more common in molars. As systemic background factors, it was significantly more frequent in patients who intake anticoagulants and/or antiplatelets and antihypertensive drugs. To prevent sudden persistent GB associated with PD, it is important to keep up with regular dental visits and maintenance of PD.
